# Recovery of Natural Polyphenols from Spinach and Orange By-Products by Pressure-Driven Membrane Processes

**DOI:** 10.3390/membranes12070669

**Published:** 2022-06-28

**Authors:** María Fernanda Montenegro-Landívar, Paulina Tapia-Quirós, Xanel Vecino, Mónica Reig, Mercè Granados, Adriana Farran, José Luis Cortina, Javier Saurina, César Valderrama

**Affiliations:** 1 Chemical Engineering Department, Escola d’Enginyeria de Barcelona Est (EEBE), Campus Diagonal-Besòs, Universitat Politècnica de Catalunya (UPC)—BarcelonaTECH, C/Eduard Maristany 10-14, 08930 Barcelona, Spain; paulina.tapia@upc.edu (P.T.-Q.); xanel.vecino@upc.edu (X.V.); monica.reig@upc.edu (M.R.); adriana.farran@upc.edu (A.F.); jose.luis.cortina@upc.edu (J.L.C.); cesar.alberto.valderrama@upc.edu (C.V.); 2 Barcelona Research Center for Multiscale Science and Engineering, Campus Diagonal-Besòs, 08930 Barcelona, Spain; 3 Chemical Engineering Department, School of Industrial Engineering—Research Center in Technologies, Energy and Industrial Processes (CINTECX), Campus As Lagoas-Marcosende, University of Vigo, 36310 Vigo, Spain; 4 Department of Chemical Engineering and Analytical Chemistry, Universitat de Barcelona, Diagonal 645, 08028 Barcelona, Spain; mgranados@ub.edu (M.G.); xavi.saurina@ub.edu (J.S.); 5 Water Technology Centre (CETAQUA), Carretera d’Esplugues, 75, 08940 Cornellà de Llobregat, Spain

**Keywords:** spinach waste, orange waste, polyphenols recovery, microfiltration (MF), ultrafiltration (UF), nanofiltration (NF), reverse osmosis (RO), integrated membrane processes

## Abstract

Spinach and orange by-products are well recognized for their health benefits due to the presence of natural polyphenols with antioxidant activity. Therefore, the demand to produce functional products containing polyphenols recovered from vegetables and fruits has increased in the last decade. This work aims to use the integrated membrane process for the recovery of polyphenols from spinach and orange wastes, implemented on a laboratory scale. The clarification (microfiltration and ultrafiltration, i.e., MF and UF), pre-concentration (nanofiltration, NF), and concentration (reverse osmosis, RO) of the spinach and orange extracts were performed using membrane technology. Membrane experiments were carried out by collecting 1 mL of the permeate stream after increasing the flow rate in 1 mL/min steps. The separation and concentration factors were determined by HPLC-DAD in terms of total polyphenol content and by polyphenol families: hydroxybenzoic acids, hydroxycinnamic acids, and flavonoids. The results show that the transmembrane flux depended on the feed flow rate for MF, UF, NF, and RO techniques. For the spinach and orange matrices, MF (0.22 µm) could be used to remove suspended solids; UF membranes (30 kDa) for clarification; NF membranes (TFCS) to pre-concentrate; and RO membranes (XLE for spinach and BW30 for orange) to concentrate. A treatment sequence is proposed for the two extracts using a selective membrane train (UF, NF, and RO) to obtain polyphenol-rich streams for food, pharmaceutical, and cosmetic applications, and also to recover clean water streams.

## 1. Introduction

In recent years, polyphenols have received great interest due to their potential as preventive and therapeutic agents in many diseases such as virus infections, allergies, and diabetes, among others [1,2]. Consequently, the global market for antioxidants is increasing rapidly due to the increasing risk to human health from being in a constantly polluted environment [3]. These agents not only have pharmaceutical applications but also cosmetic applications, with the objective of developing research at industrial and lab scales to explore the behavior of these molecules and their analogs. Therefore, there is great interest not only in their extraction, but also in the separation, purification/concentration, and recovery of antioxidant compounds from natural sources (including by-products) [4].

Several studies have shown the structure–antioxidant-activity relationship of some families of polyphenols such as hydroxybenzoic acids, hydroxycinnamic acids, and flavonoids extracted from plants [3,5]. Some flavonoids from vegetables such as spinach and from fruits such as oranges have been reported to possess a variety of biological activities and pharmacological properties, including antidiabetic, anti-inflammatory, and anticancer properties, among others [6].

Spinach and orange crops are among the most abundant in the world and are of major interest in Spain, generating between 13% and 50% of waste, respectively [6,7]. There is ample scientific evidence that spinach, oranges, and their by-products are rich sources of minerals, vitamins, and dietary fiber, as well as bioactive compounds such as polyphenols (specifically flavonoids and phenolic acids) [6,8], which also provide a high antioxidant activity [3,6]. 

The main use of spinach in the food industry is raw or cooked leaves, while oranges are used for fresh juice, juice concentrate, or orange-based drinks. The main residues generated from the processing of spinach and oranges are damaged leaves and the bagasse from the processing of spinach juice, as for orange peels and seeds. These residues can be considered an interesting source of phenolic compounds [3]. 

In the last decade, different studies have been proposed the recovery of polyphenols from spinach, but greater interest has been shown in oranges since their percentage of waste generation is higher. These natural antioxidants offer interesting perspectives and opportunities in the food industry, for example, in the production of dietary supplements and functional foods, and for their possible use by the pharmaceutical and cosmetic industries. However, the proposed methodologies have some drawbacks [9,10]. For example, extraction with organic solvents is characterized by safety problems such as toxicity, low efficiency, and time consumption. In addition, heat treatment gives rise to pyrolysis, and enzymes can be denatured in enzyme-assisted, ultrasonic, or microwave-assisted extraction. Furthermore, polyphenols are sensitive to heat, light, and oxygen exposure [3]. According to Ameer et al. [11], the antioxidant recovery from agri-food by-products is one of the most important goals.

The recovery of polyphenols from by-products can be carried out using the following main steps: (i) macroscopic pretreatment, (ii) extraction, (iii) isolation (separation of macro- and micro-molecules) and purification, and (iv) product formation [12]. 

Pressure-driven membrane processes such as microfiltration (MF), ultrafiltration (UF), nanofiltration (NF), and reverse osmosis (RO) can be used at different stages: MF in macroscopic pretreatment, UF in the separation of macro- and micro-molecules, and NF and RO in purification/concentration [3,13]. All these processes allow the concentration and separation to be carried out without using heat; another advantage is that the equipment requires little space, running costs and energy consumption are low, and products and co-products are high in quality. All these factors are very important for the recovery of by-products [14].

On the other hand, a disadvantage of membrane processes is related to the sensitivity to concentration polarization and membrane fouling due to chemical interaction with feed components, for which a pretreatment is necessary. 

Spinach and orange residues contain soluble sugars (sucrose, glucose, and fructose), insoluble carbohydrates, fibers, organic acids, essential oils, flavonoids, and carotenoids [6]. Therefore, UF can be considered a valid approach to separate and recover valuable compounds from finely divided solid materials present in the extract from the residue of these matrices, with UF being able to remove macromolecules such as pectins and proteins from the extract, ensuring the production of a clarified solution that contains beneficial compounds for health [15]. 

Cassano et al. [16,17] used UF membranes to clarify pomegranate and clementine mandarin juices containing phenols (e.g., hydroxycinnamic derivatives) without significantly affecting the antioxidant profile of the permeate, nor the nutritional or physicochemical properties.

Pinto et al. [18] evaluated the performance of different UF and NF membranes in the treatment of ethanolic extract from Eucalyptus globulus bark, obtaining rejection values for total phenolic compounds higher than for total carbohydrates, which indicates the effectiveness of UF and NF process for the removal of sugar residues in the permeate stream.

Another study, by Cassano et al. [19], evaluated UF and NF membranes in flat-sheet configurations (MWCO range 1000 to 4000 Da) to purify polyphenols from sugars in clarified pomegranate juice. The obtained results showed high retentions towards anthocyanins, total polyphenols, and total antioxidant activity (in the range of 80–95). On the other hand, the rejection values towards fructose and glucose were 1–3%, indicating the suitability of the membrane process. In addition, the authors obtained an improved purification of polyphenols by combining the concentration step with discontinuous diafiltration.

There is a great interest in the revalorization of wastes, especially those from the agri-food industry, for use as a direct source of biocompounds, such as polyphenols. First, it should be noted that these biocompounds are usually extracted by a liquid–liquid process with organic solvents. However, in this work, their extraction using water as a green extractant has been proposed. Secondly, it is important to recover these polyphenols from other impurities such as sugars and proteins, and membrane technology is proposed as a sustainable alternative to carry out a separation and concentration in a selective manner, according to the family of polyphenols found in these aqueous extracts.

Therefore, the aim of the present study is to investigate the feasibility of the membrane technology based on MF and UF as a clarification step, NF as a pre-concentration step, and RO for the concentration of polyphenols from spinach and orange aqueous extracts. Furthermore, an integrated treatment train based on membrane technologies for polyphenol separation and concentration from spinach and orange extracts is proposed.

## 2. Materials and Methods

### 2.1. Reagents

The main standards used in the experiments were 4-hydroxybenzoic acid, vanillic acid, rutin, syringic acid, gallic acid, ferulic acid, *p*-coumaric acid, and naringenin, purchased from Sigma Aldrich (St. Louis, MO, USA); kaempferol and hesperidin were obtained from Glentham Life Sciences (Corsham, UK); and caffeic acid was obtained from Chengdu Biopurify Pytochemicals (Sichuan, China). The solvents were prepared with acetonitrile (ACN, HPLC-UV grade) from Fisher Scientific (Loughborough, UK), formic acid (98–100% *w*/*w*) from Merck (Darmstradt, Germany), and water (purified with a Milli-Q equipment, Merck Millipore, Darmstradt, Germany).

### 2.2. Samples

Spinach and orange were purchased from a local market in Barcelona (Spain). One kg of each one was used in the process to simulate the waste obtained from agri-food industries, specifically in the juicing process. Spinach and orange were processed with a domestic juicer. The solid residues obtained (spinach leaf waste and orange peel and seeds) were used as representative waste samples and stored in a freezer at −20 °C.

### 2.3. Extraction Process

The optimization of operational conditions to maximize polyphenol extraction yield from spinach and orange wastes has been studied previously, and it is reported elsewhere [6]. Several parameters were evaluated, e.g., time, temperature, solid-to-solvent ratio, and pH. The optimal conditions obtained by mechanical stirring extraction were 5 min, 50 °C, 1:50 (*w*:*v*), and pH 6 for the spinach matrix; and 15 min, 70 °C, 1:100 (*w*:*v*), and pH 4 for the orange matrix.

### 2.4. Polyphenol Separation and Concentration by the Membrane Process

#### 2.4.1. Experimental Set-Up and Procedures

Experimental runs were performed using a MemHPLC cell (MMS AG Membrane Systems, Urdorf, Switzerland) with an active membrane area of 28 cm^2^. A Waters 515 HPLC pump (Waters, Milford, MA, USA) was used to pump the extract through the membrane system. Additionally, a stirring plate (Heidolph, Schwabach, Germany) placed into the feed tank was used to keep the feed stream constant.

Experiments were performed according to the batch system configuration in which the permeate stream was collected separately, while the retentate was recycled back to the feed reservoir. A variation in the feed flow from 1 mL/min to 10 mL/min was carried out, with 5 min to stabilize the system (between each feed flow); after stabilization, the permeate current was withdrawn. The obtained volume was weighed, and the flow rate of the permeate stream was calculated using the time it took to obtain it. In addition, samples were analyzed by high-performance liquid chromatography (HPLC) to determine the total phenolic content (TPC). All the experiments were performed at room temperature (25 °C). After each test, the membrane module underwent a cleaning procedure with 25 mL of Milli-Q water for 5 min at a flow rate of 5 mL/min.

The membrane performance was evaluated in terms of productivity (permeate flux) and rejection. The permeate flow rate (Q_p_), which is the flow that passes through the membrane, was calculated using Equation (1) [13]:

(1) $$Q_{p}\;\left(\mathrm{mL}/s\right)=\frac{M_{P}\;\left(g\right)}{\rho \left(g/\mathrm{mL}\right)\times t\;\left(s\right)}$$

where *Q_p_* is the permeate flow rate (mL/s); *M_p_* is the permeate collected mass (g); ρ is the density (g/mL) of the extract; and *t* is the time (s) it takes to obtain the sample.

The volumetric flux of permeate (J_v_) was calculated using Equation (2) [13]:

(2) Jv Lh×m2=QP LhA m2

where A (m^2^) is the membrane permeation area.

The rejection coefficient (R) was calculated using Equation (3) [13]:

(3) $$R\;\left(\%\right)=1-\frac{C_{P}}{C_{F}}$$

where C_p_ and C_F_ are the concentrations of solute in the permeate and in feed, respectively.

#### 2.4.2. Membrane Tests

Characteristics of the membranes used in this study are reported in Table 1. Condi-tioning of the NF and RO membranes consisted of their immersion for 12 h in Milli-Q water prior to the experiment to remove conservative products. NF and RO dense membranes must be pressurized, where the extract was pumped through the system at 10 mL/min (maximum feed flow rate). The conductivity measurements of the extracts were taken (GLP31 conductivity-meter CRISON (Barcelona, Spain)) every 10 min until two measurements were equal and the membrane was considered pressurized.

**Figure 1:**
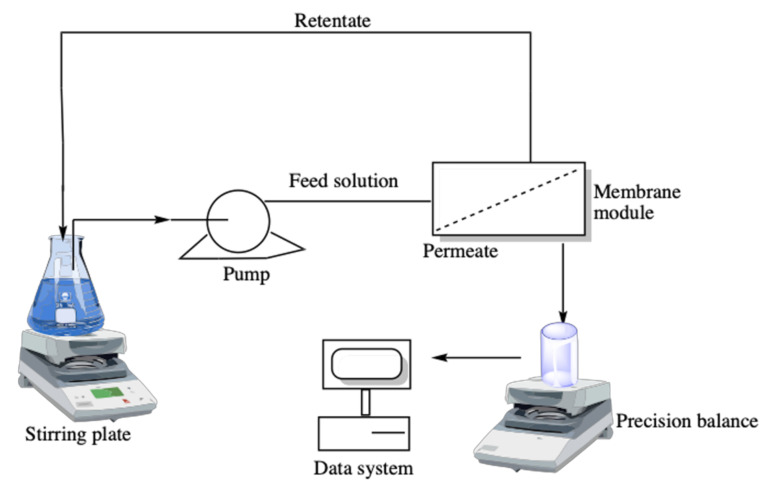
Schematic diagram of the membrane filtration process.

A 30 mL volume of the extracts from spinach and orange matrices was filtered by MF, UF, NF, and RO membranes. A general scheme of the membrane test employed in this study is shown in Figure 1.

### 2.5. HPLC Determination of TPC

The TPC was determined by HPLC with a diode array detection (DAD) equipped with a Kinetex C18 column (100 mm × 4.6 mm of internal diameter and 2.6 μm particle size, Phenomenex, Torrance, California, USA). The mobile phase was composed of 0.1% formic acid (Merck, Darmstradt, Germany, 98–100% *w*/*w*) in Milli-Q water (Merck, Darmstradt, Germany) as solvent A and ACN (Fisher Chemical, Leicestershire, UK) as solvent B. The gradient elution program was as follows: 0 min, 5% B; 30 min, 20% B; 40 min, 45% B; 40.2 min, 5% B; 50 min, and 5% B. The flow rate was 1 mL/min, and the injection volume was 5 µL. Chromatograms were recorded at 280, 310, and 370 nm. The TPC and the total hydroxybenzoic acid (HB) contents were estimated at 280 nm and expressed in terms of gallic acid equivalents (GAE) per L, the total hydroxycinnamic acid (HC) contents was determined at 310 nm and was expressed in terms of caffeic acid equivalents (CAE) per L, and the total flavonoid (F) content was estimated at 370 nm and expressed in terms of kaempferol equivalents (KE) per L [6]. 

### 2.6. Statistical Analysis

Data were analyzed by two-factor analysis of variance (ANOVA). All the membrane experiments were performed in duplicate, and results are expressed as mean ± standard derivation (SD). The *p*-values < 0.05 were considered significant.

## 3. Results and Discussions

### 3.1. Polyphenol Composition of Extracts of Spinach and Orange Wastes

The TPC in the spinach matrix at optimal extraction conditions (5 min, 50 °C, 1:50 (*w*:*v*), and pH 6) was 5.89 ± 0.88 mg/L. For the orange matrix, the TPC at the optimal extraction conditions (15 min, 70 °C, 1:100 (*w*:*v*) and pH 4) was 2.08 ± 0.07 mg/L [6]. According to Montenegro-Landívar et al. [6], for spinach waste, the main polyphenol families identified were hydroxycinnamic acids (HC, MWCO of 164.15–960.88 Da), specifically caffeic acid (0.93 ± 0.26 mg/L, MWCO of 180.15 Da) and ferulic acid (0.67 ± 0.23 mg/L, MWCO of 194.19 Da), and for the flavonoids (F, MWCO of 254.24–978.85 Da), rutin (1.47 ± 0.23 mg/L, MWCO of 610.5 Da). For orange waste, the main identified polyphenol family was flavonoids, specifically hesperidin (48.63 ± 0.85 mg/L, MWCO of 610.2 Da).

### 3.2. Performance of Selected Membranes from Spinach and Orange Extracts

Recovery of polyphenols from spinach and orange extracts were evaluated by different pressure driven membranes such as MF, UF, NF, and RO.

#### 3.2.1. Microfiltration

Spinach and orange extracts were driven towards 0.22 and 0.45 µm MF membranes, to evaluate polyphenols purification. The transmembrane flux increased when the feed flow rate increased (from 1 mL/min to 10 mL/min). This trend was found for both membranes (0.22 and 0.45 µm) and in both matrices. Therefore, the highest transmembrane flux was obtained at the maximal feed flow rate studied. Under these circumstances, the increase in J_v_ was 93% from 1 to 10 mL/min; therefore, the selected feed flow rate was 10 mL/min for spinach and orange extracts.

**Figure 2:**
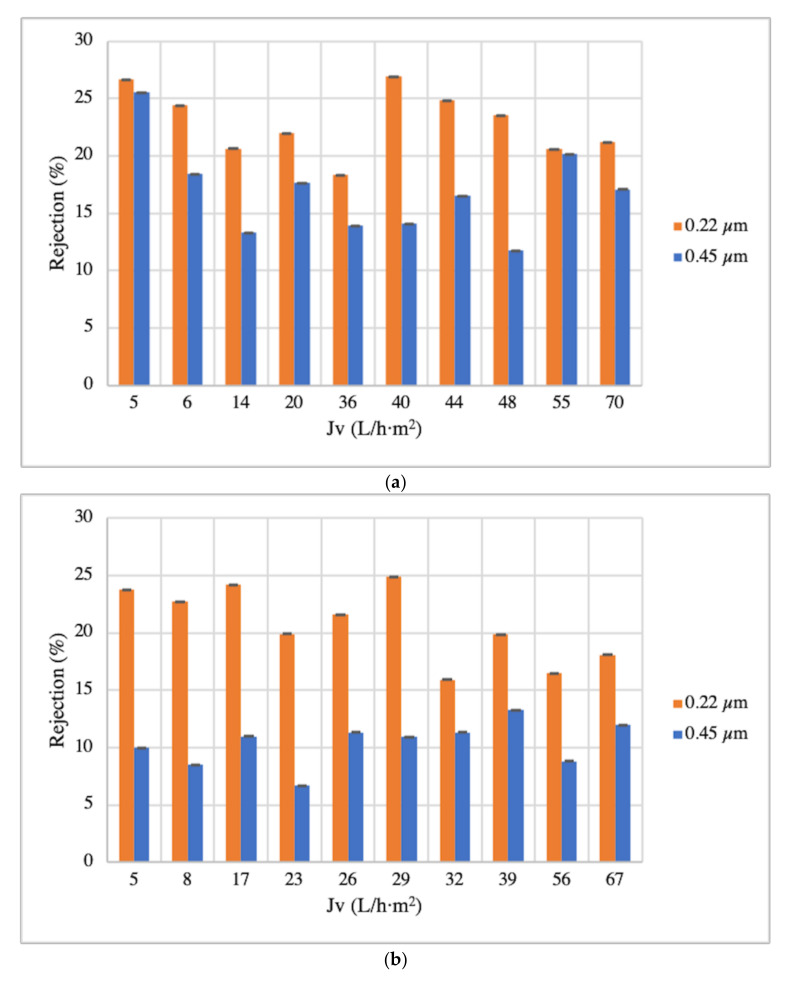
MF (0.22 and 0.45 µm) rejection evolution with permeate flux of spinach and orange matrices (**a**,**b**), respectively.

The determination of TPC in the spinach extract (5.89 mg/L) and orange extract (2.08 mg/L) revealed that the rejection of the 0.22 µm membrane (23% and 21% averages for spinach and orange matrices, respectively) was higher than the 0.45 µm membrane (17% and 10% averages for spinach and orange matrices, respectively). The ANOVA test results confirmed that there was significant difference between 0.22 and 0.45 µm membranes on the polyphenol recovery from spinach and orange waste extracts (*p* = 4.30 × 10^−6^ and *p* = 1.63^−9^, respectively; see Figure 2a,b). According to Cassano et al. [49], one of the main interactions that occur between polyphenols and MF membranes are steric exclusion and hydrophobic attraction. In fact, the rejection of MF membranes is only based on steric effects, so ions or elements will pass through them depending only on its size and its relation with the membrane’s pore size [13,50]. Thus, as shown in Table 1, both MF membranes tested were made of the same material, so one of the differences between them was the pore sizes, which were 0.22 and 0.45 µm. Additionally, the 0.45 µm membrane has a more hydrophobic surface with a contact angle value of 46.7°, while the 0.22 µm membrane is less hydrophobic with a contact angle value of 19–31°. Therefore, the large contact angle of the 0.45 µm membrane is responsible for its lower permeability (see Table 1).

The hydrophobicity of membranes is presented in terms of the contact angle between the water and membrane (contact angle value between liquid and solid). If the contact angle is higher than 90°, the membrane material is considered hydrophobic [51]. In this work, both MF membranes have contact angles lower than 90° (see Table 1). Thus, the membrane surface is less hydrophobic and more hydrophilic, so the membrane wettability rate is faster in the hydrophilic surfaces [52].

Regarding polyphenol families, the rejections of HB, HC, and F for the 0.22 µm membrane were 17%, 20%, and 24%, respectively; analogously, for the 0.45 µm membrane, they were HB 12%, HC 14%, and F 21% (see Table 2) for the spinach matrix; for the orange matrix, they were HB 0%, HC 16%, and F 24% for the 0.22 µm membrane and HB 0%, HC 10%, and F 17% for the 0.45 µm membrane (see Table 3). Therefore, the hydrophobicity of the polyphenol families (HC and F) with molecular weight (see Section 3.1) below the membrane MWCO (see Table 1) could be considered the most important parameter affecting the adsorption of phenolic compounds on the membrane surface.

With these both membranes (0.22 and 0.45 µm), low rejection percentages were expected, which indicates that the polyphenols passed through the membrane and thus were separated from solid particles and impurities. According to Comite et al. [53], MF is used especially to separate suspended solids in liquid foods. In this work, for example, for the spinach matrix, the suspended solid content of the feed (the extract) was 40 ± 0.0 mg/mL, and its content was completely removed in the permeate for the 0.22 and 0.45 µm membranes. Laorko et al. [54] also managed to remove suspended solids in addition to microorganisms by MF membranes, specifically with the 0.2 µm membrane.

The 0.22 µm membrane was selected to be the optimal membrane pore size for a clean-up stage for both matrices without losing high amounts of polyphenols (lower rejection).

#### 3.2.2. Ultrafiltration

The clarification treatments of spinach and orange extracts were evaluated with the selected UF (30 and 50 kDa) membranes.

In particular, for the spinach extracts, the 50 kDa membrane exhibited the highest permeate flux (10 mL/min), with a value of 63.16 ± 0.46 L/h·m^2^, in comparison with the permeate flux obtained by the 30 kDa membrane (60.49 ± 0.40 L/h·m^2^). In the case of the orange extracts, the 30 kDa membrane flux (55.90 ± 0.18 L/h·m^2^ (10 mL/min)) was much lower than the flux obtained with the 50 kDa membrane (77.21 ± 0.72 L/h·m^2^ (10 mL/min)). These results are in agreement with Laorko et al. [54], who obtained a lower J_v_ value with a 30 kDa than a 100 kDa membrane. They also found that the fouling phenomena could significantly affect the flux. Nevertheless, the flux decline is clearly not affected by the membrane material (see Table 1). According to Ahmad et al. [51], the flux decline can be affected by the roughness and porosity of the surface. However, the selected feed flow rate was 10 mL/min, with J_v_ improvements of 94% and 95% for the spinach and orange matrices, respectively, from 1 mL/min to 10 mL/min.

**Figure 3:**
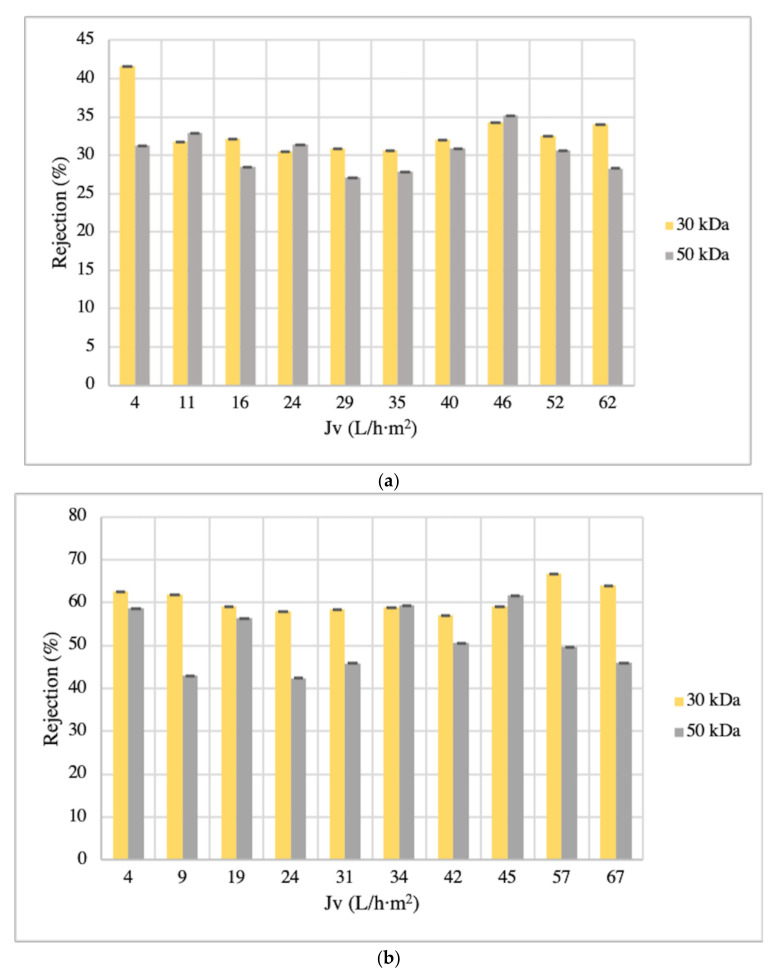
UF (30 and 50 kDa) rejection evolution with permeate flux of spinach and orange matrices ((**a**,**b**), respectively).

On the other hand, the rejection levels of total polyphenols from spinach and orange extracts for the selected membranes are also shown in Figure 3a,b, respectively.

In general terms, the greater rejection percentages of polyphenols were observed for the 30 kDa membrane in comparison with the 50 kDa membrane in both matrices’ extracts. In the case of spinach extracts, the 30 kDa membrane rejected 33% (on average), and in the 50 kDa membrane, 30% (on average) was rejected (see Figure 3a). For the orange extracts, using the 30 kDa membrane, 61% (on average) polyphenols were rejected, and with the 50 kDa membrane, 51% were rejected (on average). These results were expected, since the behavior of UF membranes can be explained based on steric consideration, and the 30 kDa membrane is the one with the lowest MWCOs (see Figure 3b). Accordingly, ANOVA assessment confirmed that there were significant differences (*p* = 0.002 for spinach and *p* = 1.24 × 10^−5^ for orange) between the two tested UF membranes (30 kDa and 50 kDa) on the polyphenol recovery.

Table 1 shows the zeta potential of 30 and 50 kDa membranes. The zeta potential is associated with how the suspension may interact with the surface of the membrane and with the possibility of the formation of films or agglomerates [25]. The zeta potential of the membranes depends on the pH. The feed pHs used in the UF process were 6 and 4 for spinach and orange extracts, respectively. In fact, at these pH values of feed (which are in the pH range; see Table 1), membranes were negative charged. The negative zeta potential values of 30 and 50 kDa membranes (−16.4 and −15, respectively) indicate higher repulsive electrostatic interactions of spinach and orange components, which could minimize fouling.

Regarding the results of three polyphenol families for the spinach matrix, the rejection percentages were 36% of HB, 32% of HC, and 40% of F for the 30 kDa membrane; the 50 kDa membrane rejected 25% of HB, 23% of HC, and 38% of F (see Table 2). On the other hand, for the orange matrix, these values were 0% of HB, 67% of HC, and 29% of F for the 30 kDa membrane; the 50 kDa membrane rejected 0% of HB, 44% of HC, and 58% of F (see Table 3). Therefore, the level of retention observed with UF membranes can be attributed to the molecular weight of hydroxycinnamic acids and flavonoids being between 164.15 and 960.88 Da and between 254.24 and 978.85 Da, respectively, and these values are lower than the nominal MWCO of the UF membranes (30,000 and 50,000 Da).

Nevertheless, focusing on the lower rejection of HC and F, the low MWCO 30 kDa membrane was selected for clarification of spinach and orange extracts. Hence, this membrane can be proposed for an integrated membrane design that represents an interesting alternative to conventional separation systems for the recovery of natural antioxidants from spinach and orange residues.

#### 3.2.3. Nanofiltration

For the polyphenol pre-concentration from the spinach and orange matrices, five membranes were studied: NF270, NF90, TFC-HR, DURACID, and TFCS (see Table 1).

For spinach and orange extracts, J_v_ values increased depending on the feed-flow rate, this trend was observed in the five membranes for the spinach extracts.

For the spinach matrix, the initial J_v_ average was 3.04 ± 0.50 (average, 1 mL/min) and increased until 32.52 ± 1.90 L/h·m^2^ (average, 10 mL/min). For the orange matrix, the permeate fluxes of NF270 and DURACID membranes were 81.53 ± 3.66 and 83.03 ± 2.54 L/h·m^2^ at 10 mL/min, respectively, which were the highest J_v_. Indeed, the DURACID membrane showed a higher permeate flux under the same operational conditions. In the case of the NF270 membrane, due to the dense polymeric structure of the rejection surface layers of this membrane, contaminants easily penetrated it, resulting in an increase in the hydraulic resistance and therefore a reduction in its permeate flux at the end of the process [55]. Regarding the DURACID membrane, the sulfonation of the hydrophilic surface of this membrane improves the biocompatibility of the polymers, favoring the permeability to water and inhibiting the adhesion of biomolecules such as proteins, and thus obtaining better permeate flux [56].

**Figure 4:**
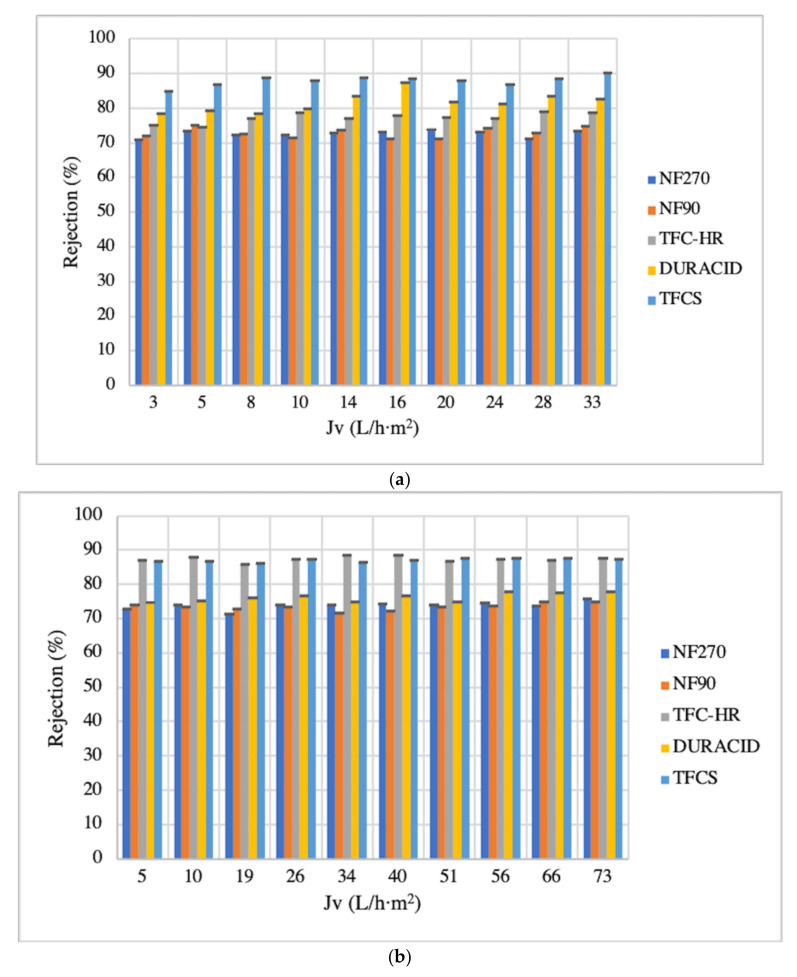
NF rejection evolution with permeate flux of spinach and orange matrices ((**a**,**b**), respectively).

Figure 4a,b show the rejection of NF membranes as a function of the permeate flux. All the NF membranes investigated presented high rejections for total phenolic content from spinach and orange extracts (rejection values higher than 70%). For the spinach matrix, the TFCS, DURACID, and TFC-HR membranes reported higher rejection values than NF90 and NF270 membranes (see Figure 4a).

For the orange extracts, TFCS (300 Da of MWCO) and TFC-HR (300–500 Da of MWCO) membranes showed the highest average rejection of 87% (see Figure 4b). Similarly, Conidi et al. [57] observed a higher rejection percentage of polyphenols (91% to 99%) by NF MWCO of 450 Da using a bergamot juice. Statistical results confirmed that significant differences (spinach matrix *p* = 8.40 × 10^−48^ and orange matrix. *p* = 8.90 × 10^−67^) were found between the studied NF membranes. Therefore, it is indispensable to consider the rejection results of the three families of polyphenols (HB, HC, and F). HB was completely rejected for all the studied membranes for both matrices. For the spinach matrix, the HC rejections were 100% for TFCS, 90% for DURACID, 83% for TFC-HR, 79% for NF270, and 78% for NF90. The F rejections were 81% for TFCS, 73% for DURACID, 71% for TFC-HR, 66% for NF90, and 63% for NF270 (see Table 2). On the other hand, for the orange matrix, the HC rejections were 89% for TFCS and TFC-HR, 79% for DURACID, 77% for NF90, and 76% for NF270. The F rejections were 72% for TFCS, 71% for TFC-HR, 66% for DURACID, 68% for NF270, and 55% for NF90 (see Table 3).

The higher rejections of polyphenols at acidic pH values (the pH values of the spinach and orange extracts were 6 and 4, respectively) can be explained by a cooperative effect of the increased interaction between the functional groups of the membrane itself, as well as the electrostatic repulsion established between the membrane-dissociated species and the charged surface of the membrane [58].

As shown in Table 1**,** the five NF membranes are made of polyamide and possess fixed dissociable carboxyl and amino groups on the surface [59]. Therefore, the pH can affect the dissociation of the membrane surface groups and the distribution charge (negative or positive) on the surface. In addition, the five membranes have similar IEPs, and also, at acidic pH, the membranes exhibit negative charge (zeta potential, Table 1).

Consequently, the electrostatic repulsion, independently of the MWCO of the selected NF membranes, contributes to the high rejection percentages of the membranes toward polyphenols from both matrices.

According to Kosmulski [60], the surface charge is determined by the existence of an isoelectric point or point of zero charge due to the presence of amino and carboxylic groups, which can dissociate in aqueous solution. The adsorption of organic compounds on the surface of the membrane can determine, in many cases, its functioning.

Another characteristic to consider is the hydrophobicity of membranes. Most high-pressure membranes are considered hydrophobic, a characteristic determined by the contact angle. The rejection and adsorption of organic solutes are not favored in membranes with higher hydrophobicity (higher contact angle) [58]. It can be seen in Table 1 that the contact angles of NF90, NF270, DURACID, TFCS, and TFC-HR membranes are 41.4–83.4°, 27–64.1°, 62.2°, 18.7°, and 35.7°, respectively. TFCS membrane has the smaller contact angle, so it has better hydrophilicity, which can more effectively prevent the membrane from being contaminated with other substances. According to Gao et al. [59], this hydrophilic behavior makes it more difficult for contaminants to be deposited, which can prolong the useful life of the membrane.

It is worth mentioning that the electrostatic repulsion also contributes to the high rejection percentages of NF membranes towards HB, HC, and F. Thus, TFCS was the membrane selected to pre-concentrate polyphenols for spinach and orange matrix extracts due to its full rejection of HB and higher rejection of HC and F. According to Montenegro-Landívar et al. [6] and Senit et al. [61], these polyphenol families report high antioxidant activity. Since spinach extracts are a suitable source of caffeic acid and ferulic acid, which are HC, and rutin, which is an F, they present values of 0.93 ± 0.26 mg/L, 0.67 ± 0.23 mg/L and 1.47 ± 0.23 mg/L amounts, respectively. Moreover, the orange extracts could be a rich source of hesperidin, an F, presenting a value of 48.63 ± 0.85 mg/L. These polyphenols could be of potential interest for cosmetic, pharmaceutical, and food applications [1,6,7].

#### 3.2.4. Reverse Osmosis

**Figure 5:**
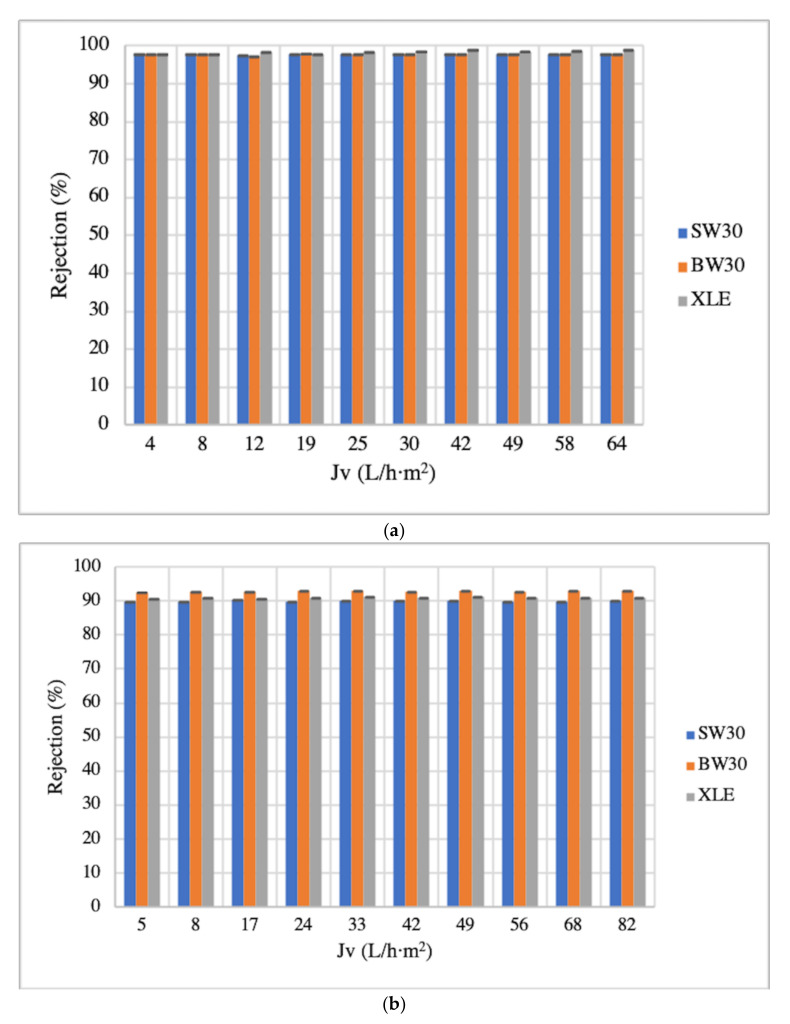
RO rejection evolution with permeate flux of spinach and orange matrices ((**a**,**b**), respectively).

For the reverse osmosis process, three membranes—SW30HR, BW30LE, and XLE—were evaluated for the concentration of polyphenols from spinach and orange extracts as shown in Figure 5.

The highest flux was obtained using the SW30HR membrane with a permeate flux of 72.67 ± 0.81 L/h·m^2^ at 10 mL/min, and the lowest permeate flux of 57.83 ± 0.73 L/h·m^2^ was obtained using the BW30LE membrane at 10 mL/min for the spinach matrix. For the orange matrix, the highest J_v_ value of 86.30 ± 1.81 L/h·m^2^ at 10 mL/min was obtained with the BW30LE membrane, followed by the permeate flux, obtaining 81.15 ± 5.48 L/h·m^2^ at 10 mL/min with the SW30HR membrane; the lowest permeate flux of 77.39 ± 0.98 L/h·m^2^ was obtained at 10 mL/min with the XLE membrane. No decline in flux was observed throughout the experiments on the selected membranes; this behavior shows that the membranes were resistant to fouling [14].

The rejection results for the three studied membranes used to treat spinach extracts (see Figure 5a) showed that the three membranes had performances ca. 100% (at 10 mL/min) of polyphenol rejection. In addition, Figure 5b shows the results of the rejection values for the orange extracts, where the BW30LE membrane displayed a higher rejection of 93% (at 10 mL/min). ANOVA results confirmed that there were significant differences between the three RO membranes for spinach and orange matrices (*p* = 2.36 × 10^−9^ and *p* = 1.82 × 10^−9^, respectively) on the polyphenol recovery.

Regarding HB, HC, and F families for the spinach matrix, the rejection results were 100% of HB, 97% of HC, and 100% of F for the XLE membrane, and 100% of HB, 93% of HC, and 100% of F for the SW30HR and BW30LE membranes (see Table 2). For the orange matrix HB, HC, and F families, HB was rejected at 100% for the three membranes; HC was rejected at 94% for BW30LE, 91% for SW30HR, and 92% for XLE; and F was rejected at 80% for BW30LE, SW30HR, and XLE (see Table 3).

The hydrophobicity of the active layer is one of the most common mechanisms used to determine if the membrane is susceptible to fouling [13]. In this case, the contact angle of the RO polyamide membranes was very similar, as can be seen in Table 1. Furthermore, the SW30HR membrane has the lowest contact angle (50–52.8°) compared to the BW30LE (50–72.2°) and XLE (55–70.9°) membranes (see Table 1). Thus, the membranes’ properties suggest that BW30LE has preferable hydrophilicity compared to the other membranes; therefore, due to its higher hydrophilicity (higher contact angle), organic solute (e.g., polyphenols) rejections were favored [58]. The XLE membrane showed higher water permeability (allowing the free passage of water to the permeate stream and concentrating polyphenols in the retentate stream [33,58]) than SW30HR and BW30LE membranes (see Table 1), since the reported pore size of the XLE (0.89 nm) membrane was higher than that of BW30LE (0.32 nm). Supplier data and the literature do not contain much information on the pore size of SW30HR membrane. According to Leo et al. [33], the surface energy, membrane roughness, and porous structure affect the water contact angle on the membrane surface.

The selected membrane for spinach extracts was XLE, due to its higher rejection of HC acids and F compared to the SW30HR and BW30LE counterparts. This is because, as has been mentioned throughout the manuscript, these polyphenol families are present in the spinach and orange wastes extracts. Thus, the selected membrane for the orange matrix was BW30LE due to the higher rejection of flavonoids. According to Gunathlikae et al. [62], the BW30 membrane can be applied to concentrate flavonoids of apple, blueberry, and cranberry juices. In addition, the antioxidant capacity of the fruit juices increased between 30% and 40%, so the BW30 membrane can be applied to enhance the bioactive concentration of fruit juices with enhanced antioxidant activity.

### 3.3. A Proposal for an Integrated Membrane Process for Polyphenol Separation and Concentration from Spinach and Orange Extracts

**Figure 6:**
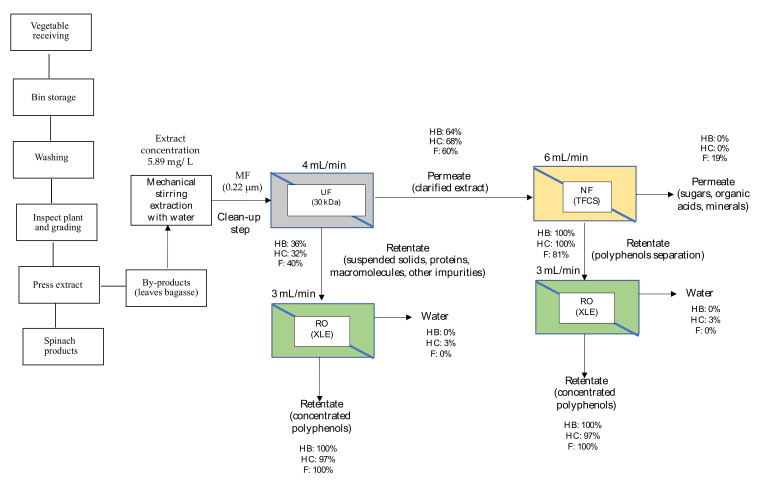
Scheme of the proposed integrated membrane process for the recovery of polyphenols from spinach extracts.

**Figure 7:**
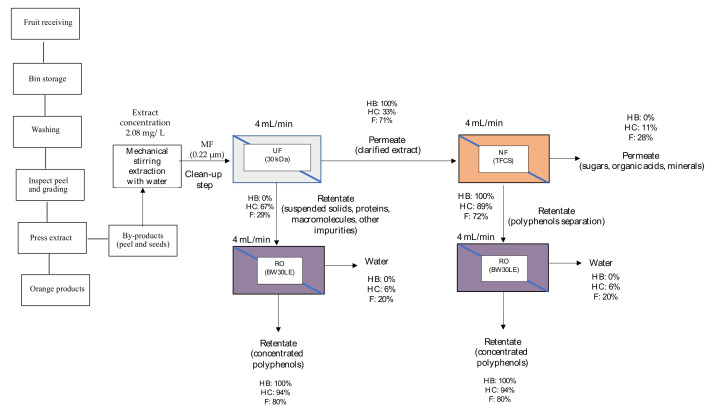
Scheme of the proposed integrated membrane process for the recovery of polyphenols from orange extracts.

An integrated treatment train is proposed for spinach and orange extracts at the selected separation conditions resulting from the membrane tests. Figure 6 and Figure 7 show the proposed schemes for the polyphenol recovery for each matrix. In these figures, the membrane type was selected based on the maximum rejection results for the three polyphenol families studied (HB, HB, and F), and the proposed operational conditions are also depicted (the feed flow rate was based on the maximum rejection results by the three polyphenol families).

It is worth mentioning that for both matrices, MF (0.22 µm membrane) is a good approach to remove suspended solids of spinach and orange extracts (cleanup stage). About the UF membranes, it can be considered the next stage that allows a complete separation of proteins, sugars, and polyphenols contained in the permeate stream. As can be seen in Figure 6 and Figure 7, 30 kDa (UF) membrane would be selected to clarify the extracts in which the three families of polyphenols are found: hydroxybenzoic acids (HB), hydroxycinnamic acids (HC), and flavonoids (F). For spinach extracts, the most abundant values are HC and F; and for orange extracts F [6], it is a priority to recover these polyphenol families.

Then, the UF permeate would be subjected to a NF process with a MWCO membrane between 150 and 300 Da. TFCS (300 Da) is proposed for spinach and orange extracts as a pre-concentration stage. Most polyphenols would be rejected by these membranes, especially HB, HC, and F. On the other hand, a permeate stream with a low polyphenol concentration would be obtained. In particular, for spinach, the NF membrane allows a permeate stream to be obtained with only F (19%), which is interesting for this matrix; for the orange matrix, the permeate stream does not contain HB, and the majority would be F (28%). Finally, the UF and NF retentate would be subjected to RO process (XLE for spinach matrix and BW30LE for the orange matrix) to concentrate polyphenols in the retentate stream. The permeate stream would be composed of water with low salinity, low COD, and reduced phytotoxicity due to the lowest contents of phenolic compounds, which are retained and concentrated by RO membranes [63]. An optional treatment for the obtained permeate could be the use of a high rejection hermetic RO technology as a second stage to polish the permeate from the first RO stage and recover pure water and a concentrated antioxidant solution with very low fouling effects. According to Konstantinos et al. [64], the results obtained with this treatment were that the proposed RO stages can be applied on a commercial scale as liquid antioxidants and water suitable for reuse purposes (e.g., beverage formulation).

For the spinach matrix, it would be possible to separate HC (3%) from HB and F in this stream, whereas for the orange extract, the permeate stream will not contain HB, and HC (6%) would be present in a low concentration, since F is the major polyphenol group (20%), which would be interesting for the orange matrix.

As a result, the three polyphenol families are concentrated in the rejection streams, making the spinach and orange extracts rich in HB, HC, and F. Additionally, the RO concentration percentages obtained with XLE were 100% for HB, 97% for HC, and 100% for F for the spinach matrix. For the orange matrix, the values for the BW30LE were HB 100%, HC 94%, and F 80%.

Conidi et al. [65] proposed a similar integrated membrane process for the fractionation and recovery of phenolic compounds. In particular, UF and NF were a valid approach for the clarification and separation, in which UF can be used as the first step to remove suspended solids from artichoke wastewater. The UF permeate can then be treated with the NP030 nanofiltration membrane (400 Da MWCO) to separate sugars from phenolic compounds, yielding a polyphenol-enriched retentate stream of interest for food, pharmaceutical, and cosmetic applications. The permeate stream is enriched in sugars of interest for food applications; this stream can be treated with the Desal DL nanofiltration membrane (MWCO 150–300 Da) to obtain water in the permeate stream, which can be reused in the artichoke industry.

Similarly, Cassano et al. [12] suggested an integrated membrane process for the recovery and concentration of flavonoids from orange press liquor, in which UF would be used as a preliminary step to remove suspended solids and obtain a flavonoid-enriched permeate stream. The UF permeate stream could be treated with NF membranes to pre-concentrate flavonoids (such as flavanones and anthocyanins), obtaining a permeate stream enriched in sugars and minerals. Finally, the NF retentate stream could be treated by OD (osmotic distillation), producing a concentrated phenolic solution of great interest for cosmetic, food, and pharmaceutical applications.

According to Conidi et al. and Cassano et al. [12,65], the proposed process allows the traditional flow chart of the spinach and orange processing industry to be redesigned with important advantages—including the reduction in decontamination costs and environmental impact, the reduction in water and energy consumption, and, above all, the recovery and reuse of high-added-value compounds such as polyphenols—compared to conventional techniques such as electrodialysis, adsorption, and desorption on macroporous resin and chromatographic techniques [65].

Galanakis et al. [66] reported that electrodialysis and NF are assumed to be safe, while adsorption and chromatographic techniques depend on the toxicity of the materials involved in the process. The cost of electrodialysis is usually higher than that of NF. However, depending on the frequency of the membrane sheet discharge, the operating cost could be very high. In addition, the efficiency of adsorbent regeneration and chromatographic column cleaning affect the cost proportionally.

Considering that pre-concentrated and concentrated extracts were produced without thermal damage to phenolic compounds, these results offer interesting prospects for the use of these products as natural colorants and/or for nutraceutical applications and a clear permeate that is reusable as process water or for membrane cleaning. For example, lignans can be used in fats and oils to increase their stability during heating and storage [67]. Plant extracts (containing polyphenols) are effectively used in water- and tea-based functional beverages [68]. Regarding pharmaceutical applications, it should be taken into account that the suggested daily intake of polyphenols is 1 g/day to provide high concentrations of metabolites in the blood [69]. Taking this suggestion into account, Rodrigo et al. [70] studied the behavior of ingested quercetin. They obtained the result that after 2 or 3 h of ingestion, quercetin was absorbed, reaching its point of maximum concentration in plasma. They also considered that the antioxidant activity was responsible for its defensive benefits by reducing the level of free radicals in the body. Additionally, in cosmetics, quercetin is used to formulate skin creams due to its high antioxidant activity [71].

## 4. Conclusions

The investigated pressure-driven membrane processes presented good potential for recovering polyphenols from spinach and orange residues for different applications such as natural antioxidants to be used as functional ingredients in the food, pharmaceutical, and cosmetic industries.

Results of this work demonstrate the suitability of MF, UF, NF, and RO to perform a purification (clean-up stage, clarification, pre-concentration, and concentration, respectively) of natural polyphenols from spinach and orange residues.

On the basis of the behavior of the transmembrane flux, these results depend on the feed flow rates of the MF, UF, NF, and RO membranes. The selected feed flow rate for both extracts was 10 mL/min for the MF, UF, NF, and RO membranes.

The 0.22 µm (MF) membrane was used to remove suspended solids from both matrices.

Concerning the UF process, the 30 kDa membrane was selected for a clarification stage for the two extracts, with rejections of 36% for hydroxybenzoic acids, 32% for hydroxycinnamic acids, and 40% for flavonoids for spinach extracts. For the orange matrix, 0% for hydroxybenzoic acids, 67% for hydroxycinnamic acids, and 29% of flavonoids were rejected.

Furthermore, NF membranes were selected for the pre-concentration stage. The best rejection percentages were obtained by using the TFCS membrane, which had a higher rejection of hydroxybenzoic acids, hydroxycinnamic acids, and flavonoids (100%, 100%, and 81%, respectively) for the spinach extracts; and for orange extracts, TFCS rejected 100% of hydroxybenzoic acids, 89% of hydroxycinnamic acids, and 72% of flavonoids.

Finally, the XLE and BW30LE (RO) membranes were selected to concentrate polyphenols from spinach and orange matrices, respectively. The rejection percentages were 100% for hydroxybenzoic acids, 97% for hydroxycinnamic acids, and 100% for flavonoids for the spinach matrix, and 100% of hydroxybenzoic acids, 94% of hydroxycinnamic acids, and 80% of flavonoids were rejected for the orange matrix.

Overall, a conceptual process design is proposed in which the MF and UF membranes could be used to obtain clean extracts from spinach and orange extracts and to remove impurities (e.g., suspended solids and microorganisms). NF membranes could be used for the pre-concentration of polyphenols from low-molecular-weight compounds. Additionally, RO membranes could be used to concentrate them and could facilitate the removal of the solvent. Concentrated polyphenols extracts (as solution and/or powder) with RO can be used as food additives for the formulation of nutraceutical products, natural colorants in cosmetics, etc.

The bottleneck of membrane processes can be the fouling, scale-up, and/or the lifetime of the membranes. However, the proposed membrane processes allow for significant advantages in comparison with other separation technologies such as the reduction in depollution costs, the environmental impact, water consumption, and the recovery of value-added compounds such as polyphenols. Overall, the valorization of agri-food wastes allows one to minimize the environmental concerns derived from its treatment and improve its use as a source of polyphenols that could be recovered by using membrane technologies in the scope of the circular economy.

## Figures and Tables

**Table 1:** Characteristics of the MF, UF, NF, and RO membranes used in this study.

Membrane		Composition	pH Range (25 °C)	T Max (°C)	P Max (bar)	Iso-Electric Point (IEP)	Contact Angle (°)	Pore Size	MWCO ^1^ (Da)	Pure Water Permeability (L/m^2^ h bar)	Zeta Potential (mV)	Reference
MF	Filter-Lab 0.22 µm	Mixed cellulose esters (MCE)	2–10	75	<1.4	5.5	31 ± 1 19	0.22 µm	>100,000	7970 ± 290 8090 ± 320 at 1 bar 3770	−21. 1 (pH 8) −9.8 (pH 8) +20 (pH 7)	[20,21]
	Filter-Lab 0.45 µm	Mixed cellulose esters (MCE)	4–8	75	<1.8	2–3.3	46.7	0.45 µm		1260 at 0.7 bar	−22.5 (pH 7)	[22,23]
UF	Biomax 30 kDa (Merck, Darmstradt, Germany)	Polyethersulfone (PES)	0–14	95	6	Around 3.5	12 ± 2.94	9.61 nm	30,000	390 ± 20	−16.4 (pH 8)	[24,25,26]
	Biomax 50 kDa (Merck, Darmstradt, Germany)	Polyethersulfone (PES)	2–13	50	0.5–3	3.05 ± 0.5	68.7 ± 2.2	100 nm	50,000	593.6 ± 84.5 at TMP: 3 bar	Around −15 (pH 7)	[27,28,29]
NF	NF90 (DuPont, Delfgauw, Netherlands)	Uncoated fully aromatic polyamide TFC ^2^	2–11	45	41	4.3 4.0	54 62.7 41.4 63.2 83.4	0.68 0.24 nm	200–400	10.6	+13 (pH 3) −7 (pH 5) −24.9 (pH 7) −28 (pH 9) −29 (pH 10)	[30,31,32,33,34]
	NF270 (DuPont, Delfgauw, Netherlands)	Uncoated semi aromatic polypiperazine amide TFC	2–11	45	41	4.5 4.1	30 27 29 64.1	0.84 nm 0.71 0.42	200–400	17.8	+7 (pH 3) −15 (pH 5) −19, −22 (pH 7) −22 (pH 9) −28 (pH 10)	[31,34,35,36]
	DURACID (Suez, Trevose, PA, USA)	Sulfonamide-based active layer and polysulfone support	<10 0–9	70	82	4.3	62.2 ± 4.2	0.47 nm	150–300	8 at TMP: 7 bar 17–32 at 15.5 bar	-	[37,38]
	TFCS (KOCH, Cansas, USA)	Proprietary TFC^®^ polyamide	4–11	45	82	3.1	18.7	-	300	49 ± 6 at 5 bar	−6.5 (pH 8)	[39]
	TFC-HR (KOCH, Cansas, USA)	Proprietary TFC^®^ polyamide	4–11	45	41	4.7	35.7	-	300–500	3.5	−9.5 (pH 7) −17 (pH 9)	[40]
RO	SW30HR (DuPont, Delfgauw, Netherlands)	Coated fully aromatic polyamide TFC	2–11	45	69	Always negative	52.8 Around 50	-	100	1.3	−17.8 (pH 10.4)	[41,42,43,44]
	BW30LE (DuPont, Delfgauw, Netherlands)	Coated fully aromatic polyamide TFC	2–11	45	41	4.2 Close to 3 4.07	72.2 59.8 55 Around 50 (>SW30HR)	0.32 nm	98 100	2.2	−12.8	[31,32,45,46]
	XLE (DuPont, Delfgauw, Netherlands)	Uncoated fully aromatic polyamide TFC	2–11	45	41	3.5	55 66.3 70.9 65.7	0.89 nm	100	8.8	+13 (pH 3) −17 (pH 5) −33 (pH 7) −38 (pH 9) −38 (pH 10)	[33,34,47,48]

^1^ MWCO: molecular weight cut-off. ^2^ TFC: Thin-film composite.

**Table 2:** Results of the rejection of each polyphenol family obtained with spinach extracts for the MF, UF, NF, and RO membranes (means of the two repetitions).

Membrane		Retentate Stream			Permeate Stream		
		HB	HC	F	HB	HC	F
MF	0.22 µm	17%	20%	24%	83%	70%	75%
	0.45 µm	12%	14%	21%	88%	86%	79%
UF	30 kDa	36%	32%	40%	64%	68%	60%
	50 kDa	25%	23%	38%	75%	77%	62%
NF	TFCS	100%	100%	81%	0%	0%	19%
	DURACID	100%	90%	73%	0%	10%	17%
	TFC-HR	100%	83%	71%	0%	17%	29%
	NF270	100%	79%	63%	0%	21%	37%
	NF90	100%	78%	66%	0%	22%	34%
RO	XLE	100%	97%	100%	0%	3%	0%
	SW30HR	100%	93%	100%	0%	7%	0%
	BW30LE	100%	93%	100%	0%	7%	0%

**Table 3:** Results of the rejection of each polyphenol family obtained with orange extracts for the MF, UF, NF, and RO membranes (means of the two repetitions).

Membrane		Retentate Stream			Permeate Stream		
		HB	HC	F	HB	HC	F
MF	0.22 µm	0%	16%	24%	100%	84%	76%
	0.45 µm	0%	10%	17%	100%	90%	83%
UF	30 kDa	0%	67%	29%	100%	33%	71%
	50 kDa	0%	44%	58%	100%	56%	42%
NF	TFCS	100%	89%	72%	0%	11%	28%
	TFC-HR	100%	89%	71%	0%	11%	29%
	DURACID	100%	79%	66%	0%	25%	34%
	NF90	100%	77%	55%	0%	24%	45%
	NF270	100%	76%	68%	0%	36%	32%
RO	BW30LE	100%	94%	80%	0%	6%	20%
	SW30HR	100%	91%	80%	0%	9%	20%
	XLE	100%	90%	80%	0%	10%	20%

## Data Availability

Data are contained within the article.
